# The Impact of Antiretroviral Therapy on Malaria Parasite Transmission

**DOI:** 10.3389/fmicb.2019.03048

**Published:** 2020-01-24

**Authors:** Raquel Azevedo, António M. Mendes, Miguel Prudêncio

**Affiliations:** Faculdade de Medicina, Instituto de Medicina Molecular, Universidade de Lisboa, Lisbon, Portugal

**Keywords:** *Plasmodium*, HIV, antiretroviral, coinfection, malaria

## Abstract

Coendemicity between the human immunodeficiency virus (HIV) and *Plasmodium* parasites, the causative agents of acquired immunodeficiency syndrome (AIDS) and malaria, respectively, occurs in several regions around the world. Although the impact of the interaction between these two organisms is not well understood, it is thought that the outcome of either disease may be negatively influenced by coinfection. Therefore, it is important to understand how current first-line antiretroviral therapies (ART) might impact *Plasmodium* infection in these regions. Here, we describe the effect of 18 antiretroviral compounds and of first-line ART on the blood and sporogonic stages of *Plasmodium berghei in vitro* and *in vivo*. We show that the combination zidovudine + lamivudine + lopinavir/ritonavir (LPV/r), employed as first-line HIV treatment in the field, has a strong inhibitory activity on the sporogonic stages of *P. berghei* and that several non-nucleoside reverse transcriptase inhibitors (NNRTI) have a moderate effect on this stage of the parasite’s life cycle. Our results expose the effect of current first-line ART on *Plasmodium* infection and identify potential alternative therapies for HIV/AIDS that might impact malaria transmission.

## Introduction

In 2018, an estimated 228 million people suffered from malaria, killing 405,000 (World Health Organization [WHO], 2019a). Malaria is caused by *Plasmodium* parasites that are transmitted to their mammalian host by the bite of female-infected *Anopheles* mosquitoes (Prudêncio et al., 2011). Sporozoites injected into the skin during a blood meal eventually reach the bloodstream and migrate to the liver, initiating the hepatic stage of the infection (Mota et al., 2001). Merozoites formed during parasite replication inside hepatocytes are released into the bloodstream, giving rise to the clinical symptoms of the disease (Prudêncio et al., 2011). When a mosquito takes a blood meal from an infected mammalian host, it ingests *Plasmodium* gametocytes that will subsequently differentiate into female and male gametes and fuse to form a zygote (Sinden et al., 2010). After 18–24 h, the zygote transforms into an ookinete, traverses the midgut epithelium, and settles in the basal lamina of the midgut wall, rounding up into an oocyst (Vinetz, 2005). During the ensuing 10–13 days, oocysts increase in size, producing thousands of sporozoites that will be released in the hemolymph and travel to the mosquito salivary glands, completing the cycle (Baton and Ranford-Cartwright, 2005; Staines and Sanjeev, 2012).

Human immunodeficiency virus (HIV) infects the immune system’s CD4^+^ T cells, inducing chronic inflammation that drives the progression into acquired immune deficiency syndrome (AIDS) (HIV/AIDS, 2019: The Basics Understanding HIV/AIDS). In 2018, 37.9 million people were reported to live with HIV, leading to an estimated 770,000 deaths in that year alone (World Health Organization [WHO], 2019b). In 2002, the World Health Organization (WHO) issued a set of guidelines to help determine the best usage of antiretroviral (ARV) compounds for the treatment of HIV-positive young adults and adolescents (World Health Organization [WHO], 2016). Since then, these guidelines have been regularly updated, and, since 2016, the recommended first-line antiretroviral therapies (ART) in adults, including pregnant women and adolescents, consist of two nucleoside reverse-transcriptase inhibitors (NRTIs) plus a non-nucleoside reverse transcriptase inhibitors (NNRTI) or an integrase strand transfer inhibitor (INSTI) (World Health Organization [WHO], 2016). The recommendation for children between 3 and 10 years old is the combination of two NRTIs with the NNRTI efavirenz (EFV), while for children under 3 years of age, a combination of the NRTI backbone with the protease inhibitors (PIs) lopinavir/ritonavir (LPV/r) is recommended (World Health Organization [WHO], 2016).

*Plasmodium* and HIV infections overlap geographically in tropical and subtropical regions, particularly in Sub-Saharan Africa, where 70% of the world’s HIV cases and 93% of the malaria cases are concentrated (Kwenti, 2018; World Health Organization [WHO], 2019a). Pregnant women, in whom *Plasmodium* infections are more severe, are at particular risk of coinfection (Kharsany and Karim, 2016; Kwenti, 2018). The outlook of either disease seems to be influenced by coinfection. On the one hand, the low CD4^+^ cell count of HIV carriers limits their immune system’s ability to mount a response against a parasite infection (Skinner-Adams et al., 2008), while on the other hand, *Plasmodium* infection can cause T-cell activation and cytokine release, which can stimulate HIV replication (Xiao et al., 1998; Skinner-Adams et al., 2008). Therefore, it is important to further understand the spectrum of activity of drugs used for treatment of either disease and their possible impact on each other.

Numerous reports describe the effect of ART on the blood and liver stages of *Plasmodium* parasites (Butcher, 1997; Skinner-Adams et al., 2004; Parikh et al., 2005; Hobbs et al., 2009, 2012; Peatey et al., 2010, 2012; Nsanzabana and Rosenthal, 2011; Machado et al., 2017). PIs have been systematically described as the most effective ARVs in inhibiting *Plasmodium* erythrocytic stages (Skinner-Adams et al., 2004; Parikh et al., 2005; Andrews et al., 2006; Lek-Uthai et al., 2008; Peatey et al., 2010; Nsanzabana and Rosenthal, 2011; Hobbs et al., 2013). Their ability to inhibit the growth of drug-susceptible and drug-resistant *Plasmodium falciparum* parasite strains has also been documented (Skinner-Adams et al., 2004; Nsanzabana and Rosenthal, 2011). The PI lopinavir (LPV) has been identified by several studies as the most potent ARV inhibiting *P. falciparum* asexual stages *in vitro* (Parikh et al., 2005; Nsanzabana and Rosenthal, 2011; Hobbs et al., 2013). The PI indinavir (IDV) has also been reported to suppress *Plasmodium cynomolgi* growth and to delay prepatency in monkeys infected with *Plasmodium knowlesi* (Li et al., 2011). *Plasmodium vivax* was found to be more sensitive to the PIs ritonavir (RTV) and saquinavir (SQV) than *P. falciparum* (Lek-Uthai et al., 2008), whereas Peatey et al. (2010) showed that ARV PIs are more active against the trophozoite and schizont stages than against the ring stages of *P. falciparum* asexual parasites in the blood. These authors also showed that the exposure of *P. falciparum* gametocyte cultures to SQV, LPV, RTV, tipranavir, and darunavir (DRV) (PIs) inhibited the formation of gametocytes–gametocytogenesis, but only tipranavir had the ability to kill gametocytes (Peatey et al., 2010). Consistent with these results, Hobbs et al. (2013) showed that prolonged drug exposure to LPV/r, LPV, and SQV reduces early- and late-stage gametocyte viability, with the latter two drugs impacting parasite exflagellation. This impairment in parasite development was also reflected on oocyst infection in the mosquito, when mosquitoes were allowed to feed on blood cultures previously treated with LPV and SQV (Hobbs et al., 2013).

The mechanism of action of PIs on *Plasmodium* is still unknown, but it has been theorized that PIs inhibit the development of malaria parasites by targeting plasmepsins (PMs) in their food vacuole (Savarino et al., 2005; Andrews et al., 2006), where they play an important role in hemoglobin degradation by *P. falciparum* (Liu et al., 2005). More recently, it has been suggested that the these drugs might also target other non-vacuolar PMs (Skinner-Adams et al., 2007; Peatey et al., 2010; Li et al., 2011; Onchieku et al., 2018). Another study suggested that treatment with PIs might affect positively the outcome of malaria infection due to an impairment of parasite sequestration by these drugs. This impairment could be explained by the deficiency in the CD36 receptor observed in some patients treated with ARVs (Nathoo et al., 2003). A recent investigation of the impact of ARVs on the liver stage of *Plasmodium* infection has shown that, consistent with what is observed for erythrocytic stages, the PIs LPV and RTV are potent inhibitors of the parasite’s development in hepatic cells (Hobbs et al., 2009). A reduction in the *in vivo Plasmodium yoelii* liver burden by NNRTIs (Hobbs et al., 2012) and of the *in vivo Plasmodium berghei* liver burden by etravirine (ETV) (Machado et al., 2017) have also been reported.

In this study, we employed a recently developed luminescence-based *in vitro* assay (Azevedo et al., 2017) to determine the ability of ARVs and current first-line ARTs to inhibit the development of *Plasmodium* mosquito stages *in vitro*. We further validated those results by assessing the *in vivo* inhibitory activity of the first-line ARTs and selected alternative drug combinations against the sexual stages of the parasite’s life cycle, as well as their impact on oocyst infection. This study demonstrates that the current field treatments against HIV have an impact on the mosquito stages of *Plasmodium* and suggest the evaluation of the possible inclusion of both rilpivirine (RPV) and ETV in alternative ARTs.

## Materials and Methods

### Animals and Parasite Lines

Six- to eight-week-old male BALB/cByJ mice were purchased from Charles River Laboratories Inc. (France). The *Pb*CSGFP-Luc (Azevedo et al., 2017) and *Pb*Fluo-frmg (Ponzi et al., 2009) *P. berghei* parasite lines were employed in the experimental work, all of which was carried out under BSL1 or ABSL2 conditions. The former parasite line expresses the fusion gene *gfp-luc* under the control of the *csp* gene promotor (RMgm-152), and the latter expresses red fluorescent protein and green fluorescent protein (GFP) under the control of stage-specific promotors for female and male gametocytes, respectively (RMgm-164). The genes were integrated by double recombination into the silent *230p* gene locus of the *P. berghei* genome.

### Ookinete Production and Culturing

Ookinetes were generated as previously described (Azevedo et al., 2017). Briefly, two male BALB/cBbyJ mice were infected with 10^7^ *Pb*CSGFP-Luc-infected red blood cells (RBCs) 3 days posttreatment with 0.1 ml phenylhydrazine (25 mg/ml). On the third day of infection, when three to six exflagellation events/field (1:4 dilution) were observed by light microscopy field (40 × magnification), mice were killed, and ∼2 ml of infected blood was collected by cardiac puncture and added to Roswell Park Memorial Institute (RPMI) 1640 Medium (Sigma) 37°C. After washing with RPMI, 5 μl of blood and 195 μl of ookinete culturing medium [RPMI-1640, 25 mM HEPES, 0.4 mM hypoxanthine, 100 mM xanthurenic acid (Fluka, 85570), 10% fetal bovine serum, pH 7.6] were added per well of a 96-well plate and incubated for 24 h at 19°C for ookinete formation. In parallel, a 1:20 dilution of blood in ookinete medium was cultured in T75 flasks for 22–24 h at 19°C for the production of ookinetes. Ookinetes were purified employing a Nycodenz (Axis-Shield) gradient. The contents of the T75 flask were collected, and the RBCs were lysed for 15 min on ice with 30 volumes of ice-cold 0.17 M ammonium chloride. After removal of the lysed RBCs by washing with RPMI-1640, ookinetes were purified on a 69% Nycodenz gradient by centrifugation at 650 × *g* and 4°C for 30 min. Following centrifugation, ookinetes were collected by aspiration of the dark brown ring formed, washed in RPMI-1640, and resuspended in 1 ml of oocyst medium [Schneider’s medium (Sigma S0146), 15% fetal bovine serum, penicillin/streptomycin (50 U/ml, 50 μg/ml), and gentamicin (50 μg/ml)].

### Oocyst Cultures

Following purification, ookinetes were cocultured with *Drosophila melanogaster* S2 cells (*Drosophila* Genomics Resource Center, Bloomington, IN, United States) in a 1:10 ratio (10^4^ ookinetes and 10^5^ S2 cells) in oocyst medium, as previously described (Azevedo et al., 2017). The cultures were maintained in 96-well plates for up to 15 days at 19°C. One quarter of the medium volume was replaced by fresh medium three times a week, and 10^5^ S2 cells were added to the medium once per week.

### Evaluation of the Activity of ARV Compounds Against *Plasmodium* Mosquito Stages *in vitro*

The activity of 10 μM of each ARV compound was independently assessed against ookinetes and oocysts. This concentration was selected based on the standards established by previous experimental work by Delves et al. (2012) and Azevedo et al. (2017) on the *Plasmodium* transmission blocking effect of various compounds and after a preliminary screen of all the compounds under evaluation at 50, 10, and 1 μM. Eighteen compounds belonging to four different classes of ARVs were evaluated: (1) PI – amprenavir (APV), atazanavir (ATV), DRV, IDV, LPV, nelfinavir (NFV), RTV, and SQV; (2) INSTIs – raltegravir (RAL); (3) NRTIs – abacavir (ABC), tenofovir (TDF), emtricitabine (FTC), zidovudine (AZT), and lamivudine (3TC); and (4) NNRTIs – ETV, nevirapine (NVP), EFV, and RPV. Drug combinations *in vitro* assays contained 10 μM of each compound. ARV compounds were obtained from the NIH AIDS and Reference Reagent Program. Ten millimolars stock solutions of the compounds was prepared in dimethyl sulfoxide (DMSO), and serially diluted compounds were employed for activity assessments. A concentration of DMSO equivalent to that present in the highest compound concentration was also used as a control in all activity assays. The compounds’ effect on gametocyte to ookinete transition was determined by adding them to 1-h-old gametocyte cultures. After 24 h, the parasite load was assessed by bioluminescence employing the Firefly Luciferase Assay Kit (Biotium) according to the manufacturer’s instructions, with some modifications. Briefly, the well contents were collected, washed with PBS, spun down, and frozen in 50 μl of 1:5 lysis buffer. Thirty microliters of the lysed supernatant was transferred into each well of a white 96-well plate. Fifty microliters of luciferin Firefly Luciferase Assay buffer (1:50 ratio) was added to the samples, and the parasite load was determined by measuring luminescence intensity using a microplate reader (Tecan Infinite M200). To assess their effect on oocyst formation, compounds were mixed with ookinetes and cultured with *D. melanogaster* S2 cells for 3 days, following which the cultures were collected and lysed, and parasite load was determined by luminescence measurement, as described above. The effect of the compounds on oocyst development was assessed by adding them to 3-day-old oocyst cultures, lysing the cultures 12 days later, and determining the parasite load by bioluminescence, as described above.

### Evaluation of the Activity of ARV Compounds Against *Plasmodium* Blood and Mosquito Stages *in vivo*

To evaluate the *in vivo* antiplasmodial activity of first-line ARV regimens and proposed modifications, three male BALB/cByJ mice per experimental group were infected with 10^7^ infected RBCs of the parasite line *Pb*Fluo-frmg from a donor previously infected from a parasite stock vial. After 24 h, and during the following 4 days, parasitemia and gametocytemia were assessed by the collection of 4 μl of tail blood in 200 μl of PBS. One hundred microliters of the solution was further diluted in PBS at a 1:1 ratio and stored at 4°C, while the remainder was diluted in a 1:1 ratio of PBS containing 1.25 mM of red fluorescent nucleic acid stain Syto^®^ 61 (ThermoFisher Scientific) and incubated for 20 min at room temperature in the dark. The samples were analyzed on an LSR Fortessa X-20 flow cytometer (Becton, Dickinson and Company). Forty-eight hours postinfection, a suspension of the compounds in sunflower oil was administered by oral gavage. Compounds were administered at an allometry-scaled dose, and in accordance with the administration regimen recommended for humans, all the compounds were administered on a 24-h schedule except for ETV, which was administered every 12 h (Supplementary Table S1). DMSO in a dosage equivalent to that present in the highest compound combination was used as a control. On the fifth day of infection, ∼50 previously starved *Anopheles stephensi* mosquitoes per experimental condition were allowed to feed for ∼30 min on anesthetized, infected, drug-treated mice. Mosquitoes were kept in standard dietary conditions, at 20°C with 80% humidity under a 12-h light/dark cycle. Ten days after infection, mosquito midguts were dissected and stained with a solution of 0.025% mercurochrome to quantify oocyst infection by microscopy analysis. Images were acquired on a Leica DM2500 and analyzed with the FIJI software (Schindelin et al., 2012).

### Statistical Analysis

Data regarding the compounds’ *in vitro* effect and mosquito infection were analyzed using the Kruskal–Wallis test. A chi-squared test was used to compare mosquito infection prevalence. Data on the compounds’ effect on parasitemia and gametocytemia *in vivo* were analyzed by non-linear regression analysis. Results were considered significant for *P* < 0.05. All statistical tests were performed using the GraphPad Prism software (version 6.00, GraphPad Software, La Jolla, CA, United States).

### Ethics Statement

All work with laboratory animals was performed according to National and European regulations (Directive 2010/63/EU). All protocols were approved by the Animal Experimentation Ethics Committee (AWB_2015_09_MP_Malaria) of the Instituto de Medicina Molecular João Lobo Antunes and are in accordance with the Federation of European Laboratory Animal Science Associations (FELASA) guidelines.

## Results

### *In vitro* Activity of ARV Compounds Against Ookinete Formation

**FIGURE 1:**
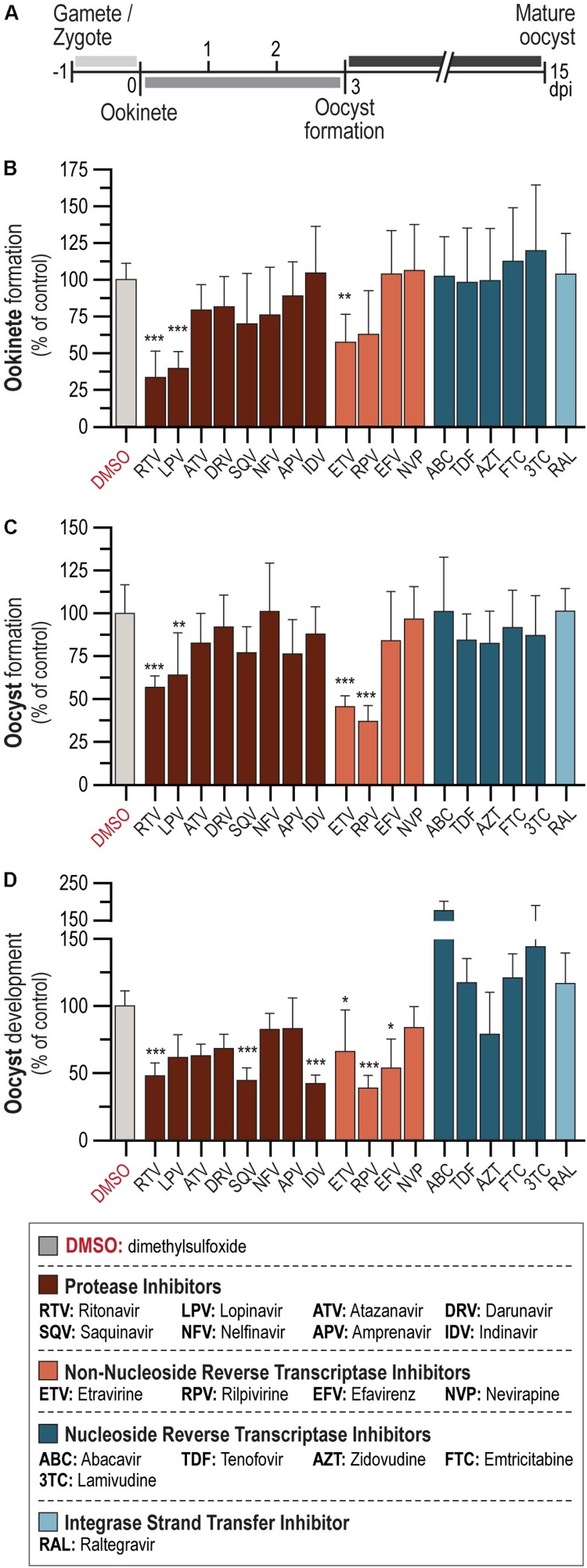
*In vitro* activity of ARV compounds on *P. berghei* sporogonic stages. **(A)** Timeline of *P. berghei* sporogonic development and drug incubation periods. **(B)** Activity of ARV compounds on the conversion of zygotes/gametes into ookinetes. **(C)** Activity of ARV compounds on oocyst formation. **(D)** Activity of ARV compounds on oocyst development. All compounds were employed at 10 μM. Bars correspond to RLU measurements represented as the percentage of RLU of the DMSO control. Results are expressed as the mean ± SD. Statistically significant differences between control and treated conditions were analyzed using the Kruskal–Wallis test. *N* = 3–6. ***P < 0.001; **P < 0.01; *P < 0.05. Detailed statistical analysis is presented in Supplementary Table S2.

The activity of a 10-μM concentration of each of 18 ARV compounds from 4 different drug classes – PI: APV, ATV, DRV, IDV, LPV, NFV, RTV, and SQV; INSTIs: RAL; NRTIs: ABC, FTC, 3TC, TDF, and AZT; and NNRTIs: EFV, ETV, NVP, and RPV – against *P. berghei* sporogonic stages *in vitro* was evaluated (Figure 1A). Our results showed that the PIs LPV and RTV led to at least 60% reduction in ookinete formation relative to the controls, whereas the NNRTI ETV inhibited parasite development by ∼40% (Figure 1B and Supplementary Table S2). Conversely, neither of the NRTI and INSTI compounds under evaluation displayed an inhibitory activity against this stage of the parasite’s sporogonic development at the concentration used in this assay (Figure 1B).

### *In vitro* Effect of ARVs on Oocyst Formation and Development

We subsequently assessed the activity of the 18 compounds listed above on oocyst formation and development. Our results showed that 10 μM concentrations of the NNRTIs RPV and ETV inhibited oocyst formation by ∼60 and 50%, respectively. A milder effect has observed for two PIs, with RTV inhibiting oocyst formation by ∼40% and LPV by ∼35% (Figure 1C and Supplementary Table S2). The PIs IDV, SQV, and RTV, as well as the NNRTI RPV, led to more than 50% inhibition of oocyst development (Figure 1D and Supplementary Table S2). Smaller effects were observed for the NNRTIs EFV and ETV, which impaired oocyst development by ∼45 and ∼30%, respectively (Figure 1D and Supplementary Table S2). Interestingly, treatment with the NRTIs ABC and 3TC consistently led to increased parasite loads relative to vehicle-treated controls (Figure 1D). None of the remaining compounds under evaluation displayed activity against either oocyst formation or development.

### *In vitro* Activity of First-Line Regimen ART Against *P. berghei* Sporogonic Stages

**FIGURE 2:**
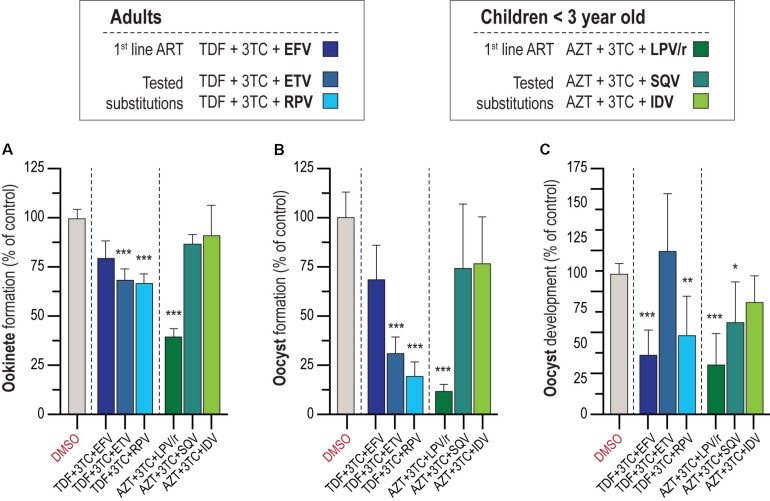
*In vitro* activity of ART on the *P. berghei* sporogonic stages. **(A)** Effect of first-line ART employed for adults and adolescents and for children under 3 years old and suggested substitutions on the conversion of zygotes/gametes into ookinetes. **(B)** Activity of first-line ART and suggested substitutions on oocyst formation. **(C)** Activity of first-line ART and suggested substitutions on oocyst development. All compounds were screened at 10 μM; TDF, tenofovir; 3TC, lamivudine; EFV, efavirenz; ETV, etravirine; RPV, rilpivirine; AZT, zidovudine; LPV/r, lopinavir/ritonavir; SQV, saquinavir; IDV, indinavir. RLU measurements represented as the percentage of RLU of the DMSO control. Statistically significant differences between control and treated conditions were analyzed using the Kruskal–Wallis test. Results are expressed as the mean ± SD. *N* = 3–4. ***P < 0.001; **P < 0.01; *P < 0.05.

According to WHO recommendations, ARV should be administered as an integral part of well-established ART regimens. The preferred backbone for first-line treatment against HIV in adults and adolescents is composed of two NRTIs and an NNRTI or INSTI, while for treatment of children <3 years old, WHO’s suggested drug combination is AZT + 3TC + LPV/r (World Health Organization [WHO], 2016). We assessed the activity of the first-line ARV regimen for adults and adolescents, TDF (NRTI) + 3TC (NRTI) + EFV (NNRTI), and children, AZT (NNRTI) + 3TC (NNRTI) + LPV/r (PIs), against the parasite’s sporogonic development (Figures 2A–C). In parallel, informed by our results regarding the activity of individual ARV compounds, we evaluated alternative drug combinations for adults and adolescents where EFV was replaced by either of the NNRTIs ETV or RPV, and alternative drug combinations for children where LPV/r was replaced by either of the best performing PIs in the individual screen, SQV or IDV (Figures 2A–C). Our results showed that a combination of 10 μM of the drugs AZT + 3TC + LPV/r displayed a ∼50% inhibitory activity against gametocyte to ookinete transition, whereas the drug combinations TDF + 3TC + ETV and RPV inhibited this process by ∼30% (Figure 2A). The combinations AZT + 3TC + LPV/r and TDF + 3TC + RPV markedly inhibited oocyst formation (∼90 and 80% inhibition, respectively), and development (∼50 and ∼40% inhibition, respectively), whereas TDF + 3TC + ETV inhibited oocyst formation by 70% but had no effect on oocyst development. Finally, the combination of TDF + 3TC + EFV also resulted in a ∼50% reduction of oocyst development but showed no effect on oocyst formation (Figures 2B,C).

### Evaluation of ART Effect on *P. berghei* Sporogonic Stages *in vivo*

**FIGURE 3:**
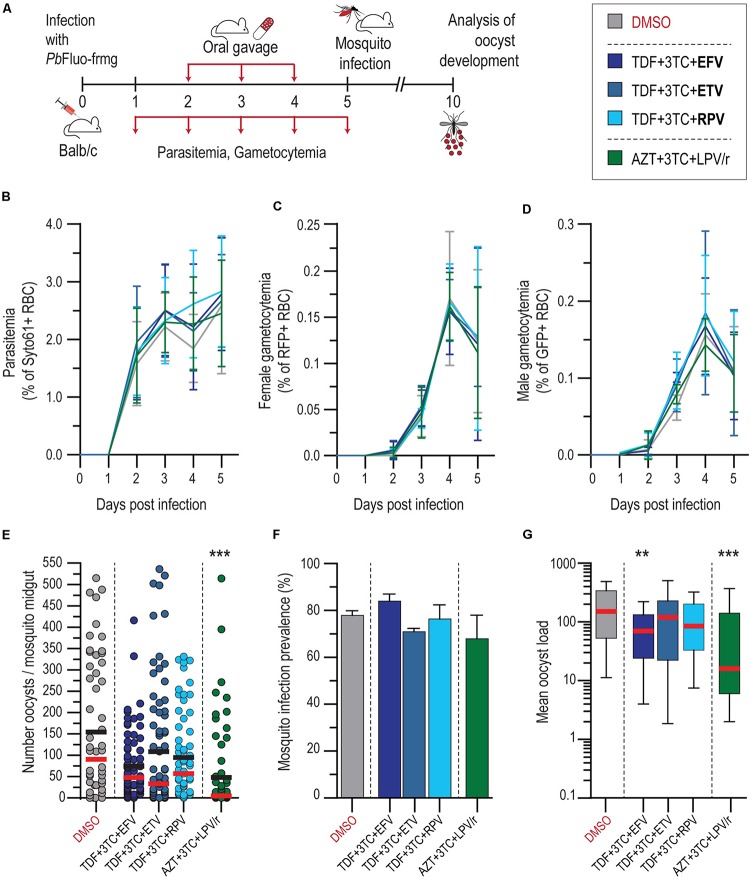
*In vivo* activity of ART on blood and transmission stages of *P. berghei*. **(A)** Schematics of drug administration and sample collection schedules. **(B–D)** Activity of ART and suggested alternative drug combinations on *P. berghei* parasitemia **(B)**, female **(C)**, and male **(D)** gametocytemia in mice. Results are expressed as the mean percentage of syto 61-positive events ± SD for parasitemia, percentage of RFP^+^ events for female gametocytemia and percentage of GFP^+^ for male gametocytemia. **(E)** Impact of ART and suggested alternatives on *P. berghei* mosquito infection measured as oocyst intensity per mosquito. Results are represented individually by number of parasites per mosquito midgut. Horizontal red and black lines represent median and mean, respectively. **(F)** Prevalence of oocyst infection in mosquitoes infected with *P. berghei* expressed as the mean ± SD. **(G)** Average *P. berghei* oocyst infection intensity upon ART and suggested alternatives in infected mosquitoes. Box plot represent the median and 25th and 75th percentile. *N* = 2. ***P < 0.001; **P < 0.01. In **(B–D)**, statistically significant differences between control and treated conditions for blood stage *P. berghei* development were analyzed using a non-linear regression analysis. In **(E)**, Kruskal–Wallis test was used to calculate *P* values and determine the significance of parasite numbers. A chi-squared test was used to compare infection prevalence values in **(F)**. Detailed statistical analysis is presented in Supplementary Table S3.

To validate our *in vitro* results, the antiplasmodial effect of the first-line drug combinations TDF + 3TC + EFV and AZT + 3TC + LPV/r, were evaluated in an *in vivo* setting (Figure 3A). Informed by our *in vitro* data, ETV and RPV were also screened in combination with TDF + 3TC. Our results showed that neither of the drug treatments employed had an impact on *P. berghei* parasitemia and gametocytemia, when compared with vehicle-treated mice (Figures 3B–D). Our data further showed that the drug combination AZT + 3TC + LPV/r displayed a strong ∼90% impact on median oocyst infection in the mosquitoes, whereas the remaining drug combinations with EFV, ETV, and RPV led to smaller reductions in the intensity of infection (Figure 3E and Supplementary Table S2). This reduction does not result from an increase in the number of non-infected mosquitoes, but rather from a reduction in the oocyst load on infected mosquitoes, which is also significant upon treatment with the TDF + 3TC + EFV combination (Figures 3F,G and Supplementary Table S3).

## Discussion

Human immunodeficiency virus and *Plasmodium* coinfections raise serious concerns in the regions where both organisms overlap geographically (Kharsany and Karim, 2016; Kwenti, 2018). It has been hypothesized that the interaction between HIV and *Plasmodium* is both synergistic and bidirectional. Thus, infection with HIV might increase the severity of *Plasmodium* infection, while the HIV viral load has been shown to increase during a *Plasmodium* infection (Nyabadza et al., 2015; Kwenti, 2018). Numerous studies report the effect of HIV ARVs on the different stages of the *Plasmodium* life cycle (Parikh et al., 2005; Andrews et al., 2006; Lek-Uthai et al., 2008; Hobbs et al., 2009, 2012, 2013; Peatey et al., 2010; Li et al., 2011; Nsanzabana and Rosenthal, 2011; Machado et al., 2017). However, little is known about how ARTs may impair the transmission and mosquito stages of *Plasmodium* parasites.

The results presented here show that the several ARV compounds impair various stages of *Plasmodium* sporogonic development *in vitro* and that the WHO-recommended first-line ARTs employed against HIV have a significant impact on *Plasmodium* infection in the mosquito vector (Figures 3E,G). However, neither of the current first-line ART nor the alternative combinations evaluated in this work inhibited *P. berghei* asexual and gametocyte stages at clinically relevant concentrations *in vivo* (Figures 3B–D). It has been shown that HIV infection leads to an increase in the production of proinflammatory cytokines tumor necrosis factor, interleuking (IL)-1β, and IL-6, which can be partially reversed by ART (Amirayan-Chevillard et al., 2000; Kedzierska and Crowe, 2001), suggesting a possible indirect effect of ART on *Plasmodium* infection. However, the lack of impact of ARVs on the blood stages of *Plasmodium in vivo* suggests that a different mechanism may be responsible for the effects observed on the parasite’s sporogony. Our results suggest that PIs might either affect the parasite’s fusing process by impairing exflagellation *in vitro*, as previously suggested for *P. falciparum* (Hobbs et al., 2013), or act further downstream of the fertilization process.

Similarly to what has previously been shown for the blood stages of *P. falciparum* (Parikh et al., 2005; Andrews et al., 2006; Peatey et al., 2010; Nsanzabana and Rosenthal, 2011; Hobbs et al., 2013), our results indicate that PIs display the strongest *in vitro* inhibitory activity against *P. berghei* transition from gametocytes to ookinetes (Figure 1B). It has been suggested that PIs act on PMs, a class of *Plasmodium*’ aspartic proteases (Parikh et al., 2005; Bonilla et al., 2007; Skinner-Adams et al., 2007; Peatey et al., 2010). Although HIV aspartic proteases are structurally different from *Plasmodium* PMs, several of the latter have been described in the sexual stages of *P. falciparum*, *P. berghei*, and *P. yoelii* (Hall et al., 2005; Young et al., 2005). Our results show that PIs strongly inhibit the formation and development of oocysts *in vitro* (Figures 1C,D). Accordingly, the first-line ART containing the PIs LPV/r had the strongest impact on oocyst intensity *in vivo* (Figures 3E,G). Although, to the best of our knowledge, the effect of ARVs on ookinetes has not been previously reported, it is known that PMs IV, VII, and X (Li et al., 2010, 2016) are expressed by this parasite stage, thus providing a possible explanation for the effect of PIs on the transformation of ookinetes into oocysts. Moreover, PM VI, whose role is yet undefined, seems crucial for the early oocyst stages of sporogonic development (Li et al., 2016). Thus, the observed inhibition of oocyst formation and development *in vitro* by PIs (Figures 1C,D) may suggest that PM VI could be the target of drugs belonging to this class.

We further observed that LPV and RTV had a stronger inhibitory activity on sporogony when tested in combination than individually (Figures 1B–D and 2A–C). RTV is currently administered exclusively as a pharmacokinetic enhancer of other PIs due to its effect on cytochrome P450 3A4 isoenzyme (Hull and Montaner, 2011). However, since this enzyme is absent from the *in vitro* system employed here, the results obtained may be explained by an additive effect of LPV and RTV. We also observed a moderate inhibition of the sporogonic stages of *P. berghei in vitro* by several NNRTIs (Figures 1A–C). A reverse transcriptase telomerase has been previously identified and characterized in *P. falciparum* (Figueiredo et al., 2005), and although it differs from HIV’s reverse transcriptase, it might contribute to explaining the observed effect of these drugs on the early and late oocyst stage of the parasite’s life cycle (Figures 1C,D).

Both the first-line ART and the alternative ARV combinations tested here had similar impacts on oocyst infection in the mosquito (Figures 3E,G). Our results show that the current first-line ART for children under 3 years old AZT + 3TC + LPV/r is the drug combination that most effectively inhibits the sporogonic stages of *P. berghei in vivo.* Interestingly, previous studies showed that LPV/r inhibits oocyst infection in *P. falciparum* (Hobbs et al., 2013) and reduces parasite *P. yoelii* liver burden (Hobbs et al., 2009). The impact of current first-line ART for adults and adolescents on mosquito infection, which includes EFV, was similar to that of the suggested alternatives employing ETV and RPV. A previous study by Machado et al. (2017) identified ETV as a stronger inhibitor of the hepatic stages of *P. berghei* than the current recommended ART with EFV.

Overall, our results suggest that both ETV and RPV, as well as other ARVs that may have an impact on *Plasmodium* transmission, should be contemplated when considering alternative ARTs in malaria-endemic regions. However, to fully ascertain the possible impact of these findings in such settings, additional work is required to assess the impact of these compounds on transmission of *P. falciparum* sporozoites. Furthermore, given the importance of the mosquito microbiota on infection by *Plasmodium*, it would be interesting to replicate our results obtained *in vivo* in mosquitoes depleted of microbiota (Dong et al., 2009; Kalappa et al., 2018). Finally, a further understanding of the mechanism of action of ARVs against *Plasmodium* parasites is essential for developing new drugs that might have both ARV and antiplasmodial activity. By identifying the target of HIV PIs on *Plasmodium* parasites, new drugs could be developed that have a stronger impact on *Plasmodium* infection.

## Data Availability Statement

The raw data supporting the conclusion of this article will be made available by the authors, without undue reservation, to any qualified researcher.

## Ethics Statement

The animal study was reviewed and approved by the Animal Experimentation Ethics Committee (AWB_2015_09_MP_Malaria) of the Instituto de Medicina Molecular João Lobo Antunes.

## Author Contributions

RA performed the experimental work. AM and MP designed and supervised the study. RA and MP wrote the manuscript. RA and AM designed the figures.

## Conflict of Interest

The authors declare that the research was conducted in the absence of any commercial or financial relationships that could be construed as a potential conflict of interest.
