# Supplementary material for: The Impact of Antiretroviral Therapy on Malaria Parasite Transmission
Source: Front Microbiol. 2020 Jan 24;10:3048. doi: 10.3389/fmicb.2019.03048 (PMC6993566; doi:10.3389/fmicb.2019.03048)
Supplement: Supplementary file 1 [file Data_Sheet_1.pdf]

Table S1. Schedules and allometry-scaled doses of antiretroviral drugs employed *in vivo*.

Drug combination	Dose in humans (w)	Dose in mice (w/W)	Schedule (h)
TDF+3TC+EFV	300 mg TDF	31.2 mg/kg TDF	24/24
	300 mg 3TC	31.2 mg/kg 3TC	24/24
	600 mg EFV	62.4 mg/kg EFV	24/24
TDF+3TC+ETV	300 mg TDF	31.2 mg/kg TDF	24/24
	300 mg 3TC	31.2 mg/kg 3TC	24/24
	200 mg ETV	20.8 mg/kg ETV	12/12
TDF+3TC+RPV	300 mg TDF	31.2 mg/kg TDF	24/24
	300 mg 3TC	31.2 mg/kg 3TC	24/24
	25 mg RPV	2.6 mg/kg RPV	24/24
AZT+3TC+LPV/r	90 mg AZT	9.9 mg/kg AZT	12/12
	45 mg 3TC	4.9 mg/kg 3TC	12/12
	120 mg LPV	13.1 mg/kg LPV	12/12
	20 mg RTV	2.2 mg/kg RTV	12/12

Table S2. Evaluation of ARV compounds' activity against *P. berghei* mosquito stages *in vitro*. Additional statistical data regarding the results presented in Fig.1B-D.

		PIs								NNRTIs				NRTIs					INSTI
**Ookinete Formation**	**DMSO**	**RTV**	**LPV**	**ATV**	**DRV**	**SQV**	**NFV**	**APV**	**IDV**	**ETV**	**RPV**	**EFV**	**NVP**	**ABC**	**TDF**	**AZT**	**FTC**	**3TC**	**RAL**
**N replicate wells**	18	8	12	9	9	11	8	9	9	9	9	9	8	8	12	8	9	8	9
**Median**	102.9	24.9	36.5	79.2	84.8	82.8	72.6	88.7	86.6	52.7	51.5	99.2	105.7	96.4	96.8	86.3	100.6	116.4	93.7
**Percentile**	87.7 - 109.5	21.0 - 54.6	30.9 - 47.4	63.5 - 93.9	71.2 - 97.2	34.7 - 102.8	57.1 - 107.1	63.7- 112.5	78.0 - 138.3	42.6 - 79.5	38.9 - 97.1	85.7 - 120.5	87.8 - 122.0	82.0 - 111.8	61.9 - 136.8	70.6 - 140.3	82.3 - 146.4	99.3 - 146.8	89.4 - 136.6
**% Difference from Median**		-78.0	-66.4	-23.7	-18.2	-20.1	-30.3	-14.2	-16.3	-50.2	-51.4	-3.7	+2.8	-6.5	-6.1	-16.6	-2.3	+13.5	-9.2
**Mean**	100.0	33.2	39.5	79.3	81.5	69.8	75.9	88.9	104.5	57.3	62.7	103.9	106.3	102.3	98.2	99.3	112.3	119.8	103.7
**Std. Dev.**	11.1	18.1	11.4	17.3	20.6	34.4	32.5	23.2	31.8	19.1	29.8	29.5	31.2	27.0	37.0	35.5	36.6	31.2	27.8
**% Difference from Mean**		-66.8	-60.5	-20.7	-18.5	-30.2	-24.1	-11.1	+4.5	-42.7	-37.3	+3.9	+6.3	+2.3	-1.8	-0.7	+12.3	+19.8	+3.7
**Kruskal-Wallis test p-value**		0.0001 (****)	0.0001 (****)	ns	ns	ns	ns	ns	ns	0.007 (**)	ns	ns	ns	ns	ns	ns	ns	ns	ns

		PIs								NNRTIs				NRTIs					INSTI
**Oocyst Formation**	**DMSO**	**RTV**	**LPV**	**ATV**	**DRV**	**SQV**	**NFV**	**APV**	**IDV**	**ETV**	**RPV**	**EFV**	**NVP**	**ABC**	**TDF**	**AZT**	**FTC**	**3TC**	**RAL**
**N replicate wells**	24	9	12	9	12	12	10	12	12	9	12	11	10	7	9	9	7	12	6
**Median**	100.1	60.5	59.4	92.5	96.1	76.4	109.6	82.4	91.8	45.8	38.5	81.6	94.2	112.9	88.2	77.1	96.1	91.1	104.0
**Percentile**	91.3 - 107.0	51.2 - 61.9	42.9 - 87.15	68.7 - 95.5	89.7 - 99.8	63.4 - 91.1	76.1 - 122.5	57.8 - 89.8	78.4 - 97.4	39.5 - 52.5	27.7 - 46.1	65.3 - 113.5	82.9 - 112.1	73.6 - 113.4	70.6 - 95.2	71.0 - 85.8	68.4 - 108.8	62.6 - 108.0	86.2 - 111.6
**% Difference from Median**		-39.6	-40.7	-7.6	-4.1	-23.7	+9.5	-17.7	-9.02	-54.3	-62.1	-18.5	-5.8	+12.8	-11.88	-23.0	-4.03	-8.9	+3.9
**Mean**	100	57.0	64.2	82.7	92.0	77.0	101.2	76.4	88.0	45.7	37.1	84.1	96.7	101.2	84.5	82.5	91.7	87.1	101.3
**Std. Deviation**	16.7	6.6	24.5	17.3	18.6	15.1	28.0	20.0	15.8	6.4	9.2	28.7	18.9	31.6	15.2	18.8	21.8	23.2	13.2
**% Difference from Mean**		-42.9	-35.8	-17.3	-7.9	-22.9	+1.2	-23.6	-12.0	-54.4	-62.9	-15.8	-3.2	+1.2	-15.5	-17.6	-8.2	-12.8	+1.3
**Kruskal-Wallis test p-value**		0.0002 (***)	0.002 (**)	ns	ns	ns	ns	ns	ns	0.0001 (****)	0.0001 (****)	ns	ns	ns	ns	ns	ns	ns	ns

Table S2 (continued). Evaluation of ARV compounds' activity against *P. berghei* mosquito stages *in vitro*. Additional statistical data regarding the results presented in Fig.1B-D.

		PIs								NNRTIs				NRTIs						INSTI
**Oocyst Development**	**DMSO**	**RTV**	**LPV**	**ATV**	**DRV**	**SQV**	**NFV**	**APV**	**IDV**	**ETV**	**RPV**	**EFV**	**NVP**	**ABC**	**TDF**	**AZT**	**FTC**	**3TC**	**RAL**
**N replicate wells**	18	11	11	9	7	11	9	8	12	15	12	9	9	9	6	6	8	11	12
**Median**	97.6	51.3	68.8	60.5	67.4	42.5	77.6	78.0	40.3	76.6	38.7	54.5	82.3	175.3	115.4	78.8	125.0	127.4	121.5
**Percentile**	93.0 - 109.6	43.5 - 54.9	42.7 - 69.9	56.2 - 69.6	59.5 - 79.6	36.8 - 50.3	73.2 - 92.2	61.5 - 109.4	36.4 - 46.9	36.0 - 91.4	32.5 - 44.0	36.4 - 60.9	68.3 - 100.5	156.2 - 196.6	100.7 - 136.7	53.3 - 109	110.3 - 133.3	102.2 - 191.3	116.6 - 132.1
**% Difference from Median**		-46.3	-28.7	-37.1	-30.2	-55.1	-20.0	-19.6	-57.3	-20.9	-58.8	-43.1	-15.3	+77.7	+17.8	-18.8	+27.4	+29.8	+23.9
**Mean**	100	47.9	61.5	62.8	68.3	44.5	82.3	83.0	42.0	66.0	38.7	53.6	83.8	175.9	117.4	78.8	120.8	144.0	116.7
**Std. Dev.**	11.1	9.6	17.1	8.5	10.6	9.2	12.1	22.9	6.4	30.9	9.4	21.6	15.8	28.9	17.9	31.3	18.0	46.2	22.8
**% Difference from Mean**		-52.1	-38.5	-37.1	-31.7	-55.5	-17.7	-16.9	-57.9	-33.9	-61.2	-46.3	-16.2	+75.9	+17.4	-21.1	+20.8	+44.0	+16.7
**Kruskal-Wallis test p-value**		0.0006 (***)	ns	ns	ns	0.0001 (***)	ns	ns	0.0001 (****)	0.0408 (*)	0.0001 (****)	0.0108 (*)	ns	ns	ns	ns	ns	ns	ns

Table S3. Evaluation of ARV compounds' activity against *P. berghei* mosquito stages *in vivo*. Additional statistical data regarding the mosquito infection results presented in Fig. 3E-G.

	DMSO	TDF+3TC+EFV	TDF+3TC+ETV	TDF+3TC+RPV	AZT+3TC+LPV/r
n (with zeros)	54	69	79	61	68
Range (with zeros)	0-515	0-416	0-536	0-331	0-514
Median (with zeros)	90.5	48.0	33.0	57.0	5.0
Percentile (25%-75%)	9.7-310.3	14.0-115.5	0.0-170.0	8.0-160.5	0.0-36.5
% decreased median		47.0%	63.5%	37.0%	94.5%
Mean (with zeros)	155.5	75.0	108.2	95.4	49.5
Std. Deviation	162.6	82.4	145.3	103.5	99.4
% decreased mean		51.8%	30.4%	38.7%	68.2%
Kruskal-Wallis test p-value		ns	ns	ns	<0.0001 (***)
Prevalence	77.9%	84.3%	71.4%	76.4%	68.0%
Std. Deviation	2.0%	3.1%	1.4%	6.85%	10.3%
Chi-square test p-value		ns	ns	ns	ns
n (without zeros)	42	59	56	49	43
Range (without zeros)	6-515	1-416	1-536	3-331	1-514
Median (without zeros)	150.5	70.0	120.0	85.0	16.0
Percentile (25%-75%)	52.7-339.8	24.0-132.0	22.2-227.5	33.0-202.0	6.0-141.0
% decreased median		53.5%	20.3%	43.5%	89.4%
mean (without zeros)	199.9	87.7	152.6	118.7	78.3
Std. Deviation	158.4	82.6	151.8	102.8	116.0
% decreased mean		56.1%	23.7%	40.6%	60.8%
Kruskal-Wallis test p-value		<0.01 (**)	ns	ns	<0.0001 (***)
